# Clinicopathologic Disease Characteristics and Their Association with Adjuvant Chemotherapy Outcomes in Pulmonary Large-Cell Carcinoma Patients with or Without Neuroendocrine Features

**DOI:** 10.3390/diagnostics15202582

**Published:** 2025-10-13

**Authors:** Doğan Bayram, Oznur Bal, Efe Cem Erdat, Serhat Sekmek, Saliha Yılmaz, Perihan Perkin, Süleyman Gökalp Güneş, Efnan Algin, Bülent Mustafa Yenigün

**Affiliations:** 1 Department of Medical Oncology, Ankara City Hospital, 1604 Street, No. 9, 06000 Ankara, Turkey; dr_ozn@yahoo.com (O.B.); serhatsekmek@gmail.com (S.S.); perihanperkin@gmail.com (P.P.); efnanalgin@gmail.com (E.A.); 2 Department of Medical Oncology, Ankara Gülhane Training and Research Hospital, 06010 Ankara, Turkey; cemerdat@gmail.com; 3 Department of Thoracic Surgery, Faculty of Medicine, Ankara University, 06230 Ankara, Turkey; drsalihaylmz@gmail.com (S.Y.); gokalpgunes@yahoo.com (S.G.G.); drbulent18@hotmail.com (B.M.Y.)

**Keywords:** large-cell carcinoma, large-cell neuroendocrine carcinoma, adjuvant chemotherapy

## Abstract

**Background:** Large-cell carcinoma (LCC) and large-cell neuroendocrine carcinoma (LCNEC) are kinds of rare lung tumors classified as distinct forms of non-small-cell lung cancer (NSCLC). They both differ in cellular morphology, neuroendocrine marker expression, and clinical outcomes. Thus, LCC and LCNEC exhibit different clinicopathological characteristics and survival outcomes. This study seeks to assess how clinicopathological and immunohistochemical features influence the need for adjuvant chemotherapy in individuals with early-stage, surgically resected LCC or LCNEC. **Methods:** This multicenter retrospective analysis included 79 patients who underwent surgical resection for large-cell carcinoma (LCC) or large-cell neuroendocrine carcinoma (LCNEC) between January 2008 and March 2025. We evaluated prognostic factors that influence survival in patients with LCC and LCNEC and assessed the effect of adjuvant chemotherapy on survival outcomes. **Results:** This study included 79 patients—39 diagnosed with LCC and 40 diagnosed with LCNEC. All patients were in stages I–III and received curative surgery. The median age was 61 years for LCC patients and 58.5 years for LCNEC patients. The median overall survival (mOS) was 80.1 months for patients with LCC and 34.2 months for those with LCNEC. Multivariate Cox regression analysis revealed that age (HR: 0.279), stage (HR: 0.198), and chromogranin A expression (HR: 0.088) were independent prognostic factors for overall survival in LCC patients. In LCNEC patients, stage (HR: 0.20), synaptophysin expression (HR: 0.30), type of surgery (HR: 0.31), and adjuvant chemotherapy (HR: 0.264) were identified as factors influencing overall survival. Adjuvant chemotherapy improved overall survival in LCNEC patients (67.0 vs. 17.8 months). **Conclusions**: Patients with LCNEC generally have poorer prognoses than those with LCC, exhibiting reduced overall survival periods. Disease stage is the most significant factor influencing overall survival for both groups. Notably, in LCNEC patients, adjuvant chemotherapy was found to independently improve survival outcomes regardless of stage.

## 1. Introduction

Lung cancer remains a primary contributor to cancer-related mortality globally, exhibiting a range of histological subtypes. Notably, large-cell carcinoma (LCC) and large-cell neuroendocrine carcinoma (LCNEC) are both classified as non-small-cell lung cancers (NSCLCs) and represent significant entities within this spectrum. LCC presents with tumor cells that have abundant cytoplasm and large, vesicular nuclei, while LCNEC is identified by its neuroendocrine morphology, as well as its expression of neuroendocrine markers [1].

LCC is characterized by poor differentiation and morphological heterogeneity, and it may display squamous, glandular, and neuroendocrine features immunohistochemically [2]. While large-cell carcinomas typically do not display neuroendocrine differentiation, they can sometimes express neuroendocrine markers such as chromogranin A, synaptophysin, and CD56. LCC occurs more frequently in older males who smoke and is associated with a prognosis that is less favorable than that of other non-small-cell lung cancers. Contributing clinicopathological characteristics include larger tumor size, recurrent necrosis, and elevated Ki-67 expression [3].

Large-cell neuroendocrine carcinoma (LCNEC) is a rare subtype of lung malignancy, accounting for approximately 0.3% to 3% of all lung cancer cases. LCNEC shares epidemiological characteristics with large-cell carcinoma (LCC) and most frequently affects male patients with a history of tobacco use. The diagnosis of LCNEC is established through neuroendocrine morphological features and immunohistochemical detection of at least one neuroendocrine marker CD56, synaptophysin, or chromogranin A in no less than 10% of tumor cells [4]. LCNEC demonstrates greater aggressiveness compared to other non-small-cell lung cancers and follows a clinical trajectory similar to that of small-cell lung cancer. The tumors typically localize peripherally, often within the upper lobes. Due to its high mitotic index, LCNEC is frequently identified at an advanced, metastatic stage. Notably, recurrence rates are substantial, even in cases that are detected early and managed by surgical resection [5].

According to the current international guidelines, the management of resected non-small-cell lung cancer (NSCLC) is primarily based on tumor stage rather than histological subtype. According to the ESMO 2025 guidelines, adjuvant cisplatin-based chemotherapy is the standard of care following complete resection in stage II–III NSCLC. Meanwhile, this form of chemotherapy is not recommended for stage IA disease, and its benefits are unknown in stage I disease displaying high-risk features (e.g., visceral pleural invasion, lymphovascular invasion, or high-grade histology); no specific recommendations are available for LCC or LCNEC due to their rarity [6]. Similarly, the 2024 NCCN Guidelines for NSCLC recommend adjuvant chemotherapy in stage II–IIIA disease while noting that evidence for uncommon histologies such as LCC and LCNEC is limited and largely extrapolated from other NSCLC subtypes [7]. Consequently, there is no consensus regarding the role of adjuvant chemotherapy in these rare tumors, highlighting the need for retrospective analysis and real-world data to better define optimal treatment strategies.

The aim of this study was to evaluate the prognostic impact of clinicopathological and immunohistochemical features in patients with surgically resected LCC and LCNEC. In particular, we sought to clarify the role of adjuvant chemotherapy in these two rare histological subtypes, for which current guidelines provide no specific recommendations. By analyzing survival outcomes in LCC and LCNEC separately, our goal was to determine whether adjuvant chemotherapy confers differential benefit across tumor types and stages; thus, this study contributes real-world evidence to help guide clinical decision-making in this underexplored area.

## 2. Materials and Methods

This retrospective study was conducted at two reference medical centers in Ankara, Türkiye. All patients diagnosed with LCC or LCNEC between January 2008 and March 2025 were included in this study. All patients were over the age of 18. Clinicopathological data, including age, gender, smoking status, disease stage, pathological characteristics, and treatment details, were collected and analyzed for each patient. The investigators retrospectively collected all data from electronic medical records and systematically reviewed all medical records to extract demographic, clinical, pathological, and treatment-related information. In our study, no patients who had perioperative complications, synchronous small-cell carcinoma, or pathological patterns other than large-cell carcinoma were included, and all patients underwent curative surgery.

At the participating centers, patient management generally involved a multidisciplinary team of medical oncologists and thoracic surgeons, and, in one of the two centers, radiation oncologists; however, not all cases were discussed by a tumor board. The staging of patients was based on postoperative pathological findings and followed the criteria outlined in the 8th edition of the American Joint Committee on Cancer (AJCC) Staging Manual [8].

In our study, immunohistochemical (IHC) evaluation included TTF-1, P40, synaptophysin, chromogranin A, and CD56 detection. For neuroendocrine markers, positivity was generally defined as immunoreactivity in ≥10% of tumor cells. IHC data were retrospectively obtained from postoperative pathology reports, and each marker was documented as positive or negative according to the original diagnostic interpretation of institutional pathologists.

Overall survival (OS) refers to the period from a patient’s disease onset to their death by any cause or to their last follow-up. Disease-free survival (DFS) is defined as the duration from a patient’s diagnosis to either a relapse or their death by any cause.

### Statistical Analysis

The statistical analyses were performed using SPSS version 22.0 (IBM Corp., Armonk, NJ, USA). Descriptive statistics are presented as counts and percentages for categorical variables and as medians and IQRs for continuous variables. Continuous variables were analyzed using the Mann–Whitney U test, while categorical variables were assessed with Pearson’s Chi-squared test or Fisher’s exact test, as appropriate. Survival analysis was performed using the Kaplan–Meier method and the Log-rank test. Multivariable analysis employed the Cox proportional hazards model. Adjusted hazard ratios (HRs), 95% confidence intervals (CIs), and *p*-values were reported; significance was set at *p* < 0.05.

## 3. Results

### 3.1. Patient Characteristics

This study comprised 39 patients diagnosed with LCC and 40 patients diagnosed with LCNEC; these patients had similar median ages of 61 and 58 years, respectively. The cohorts were predominantly male, representing 84.6% and 87.5% of each group, respectively. Most individuals reported a history of tobacco use. Postoperative pathological assessment revealed higher rates of synaptophysin, chromogranin, and CD56 positivity among LCNEC patients. A summary of the clinicopathologic characteristics is presented in Table 1.

### 3.2. Treatment Modalities

Lobectomy was carried out in 71.8% of patients diagnosed with LCC and in 85% of those with LNEC. Adjuvant chemotherapy was given to 72.5% of LNEC patients and 56.4% of LCC patients. The cisplatin/vinorelbine regimen was used most frequently for LCC, while cisplatin/etoposide was most commonly administered for LNEC. Adjuvant radiotherapy was provided to 22.5% of LNEC patients and 10.3% of LCC patients. Table 2 provides an overview of the treatment approaches for these patient groups.

### 3.3. Survival Analysis and Treatment Effect—LCC Patients

The median overall survival (mOS) among the 39 patients diagnosed with LCC was 80.1 months, with a median follow-up period of 86.3 months. Univariate analysis revealed no statistically significant associations between overall survival and variables such as gender, smoking history, tumor localization, TTF-1, P40, synaptophysin, CD56, adjuvant radiotherapy, or type of surgery. However, age, chromogranin A, and disease stage demonstrated statistically significant correlations with overall survival according to univariate analysis. Further, Cox regression analysis identified age (*p* = 0.047, HR: 0.279, 95% CI: 0.079–0.984), stage (*p* = 0.016, HR: 0.198, 95% CI: 0.053–0.741), and chromogranin A expression (*p* = 0.011, HR: 0.088, 95% CI: 0.013–0.577) as independent prognostic factors for overall survival. Comprehensive details of the survival analysis are presented in Table 3.

The median disease-free survival (DFS) for patients with large-cell carcinoma was 77.14 months. Univariate analysis did not identify a statistically significant relationship between DFS and variables including age, gender, smoking history, tumor localization, TTF-1, P40, chromogranin A, synaptophysin, adjuvant chemotherapy, adjuvant radiotherapy, or type of surgery. Disease stage and CD56 were associated with DFS at a statistically significant level according to the univariate analysis. Cox regression analysis showed that stage (*p* = 0.022, HR = 0.26, 95% CI = 0.084–0.823) and CD56 (*p* = 0.034, HR = 0.35, 95% CI = 0.136–0.926) function as independent prognostic factors for DFS. The survival analysis results are shown in Table 3.

**Figure 1:**
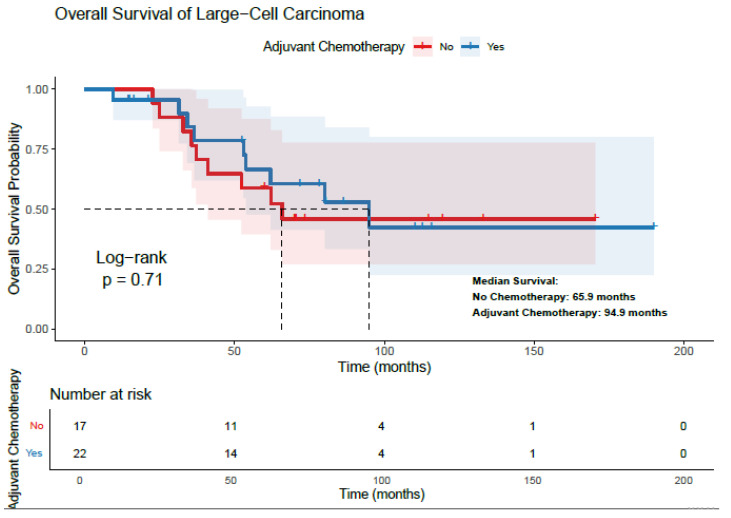
Relationship Between Adjuvant Chemotherapy and Overall Survival in Patients with Large-Cell Carcinoma.

In the overall LCC cohort, patients who received adjuvant chemotherapy had a longer median OS compared to those who did not receive it (94.9 vs. 65.9 months), although this difference was not statistically significant (*p* = 0.71) (Figure 1). In subgroup analyses, however, adjuvant chemotherapy was associated with a significant survival benefit in stage II–III LCC patients, with a median OS of 80.1 vs. 32.0 months (*p* = 0.009) (Table 4). Table 4 displays information regarding the relationship between adjuvant chemotherapy and survival within different LCC patient subgroups.

### 3.4. Survival Analysis and Treatment Effect—LCNEC Patients

For the 40 LCNEC patients, the median overall survival was 34.2 months with a 65.7-month median follow-up. Univariate analysis showed no significant association between overall survival and gender, smoking, tumor site, TTF-1, CD56, or adjuvant radiotherapy. However, stage, chromogranin A, synaptophysin, adjuvant chemotherapy, and surgery type were significantly related to survival.

Cox regression analysis was used to identify stage (*p*: 0.017, HR: 0.20, 95% CI: 0.061–0.773), synaptophysin (*p*: 0.037, HR: 0.30, 95% CI: 0.097–0.929), type of surgery (*p*: 0.048, HR: 0.31, 95% CI: 0.104–0.959), and adjuvant chemotherapy (*p*: 0.030, HR: 0.264, 95% CI: 0.079–0.880) as independent prognostic factors for overall survival. Details of the survival analysis are provided in Table 5.

The median disease-free survival (mDFS) for patients diagnosed with large-cell neuroendocrine carcinoma was 26.71 months. Univariate analysis did not reveal a statistically significant association between mDFS and age, gender, smoking history, tumor localization, TTF-1, chromogranin A, synaptophysin, CD56, adjuvant radiotherapy, or type of surgery. Stage, type of surgery, and adjuvant chemotherapy were associated with statistically significant relationships with mDFS in univariate analysis.

Further Cox regression analysis determined that stage (*p*: 0.005, HR: 0.20, 95% CI: 0.065–0.617) and adjuvant chemotherapy (*p*: 0.02, HR: 0.21, 95% CI: 0.078–0.557) were independent prognostic factors for disease-free survival. Survival analysis outcomes of LCNEC are presented in Table 5.

**Figure 2:**
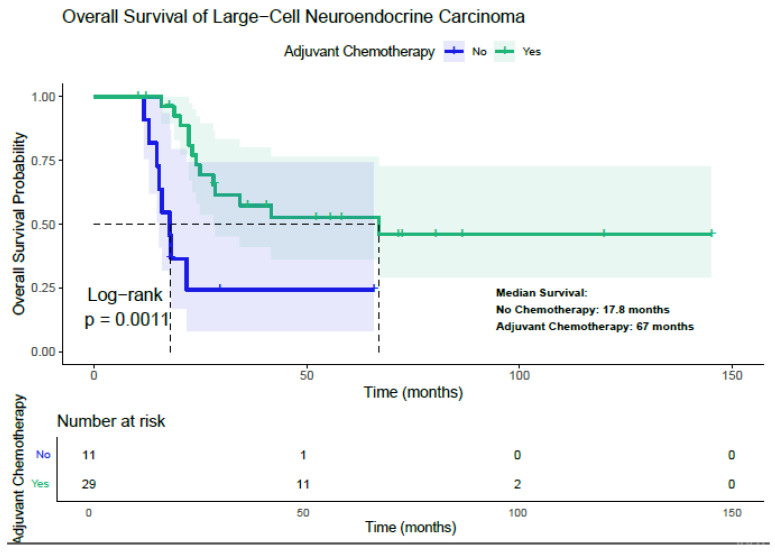
Relationship Between Adjuvant Chemotherapy and Overall Survival in Patients with LCNEC.

Adjuvant chemotherapy was found to significantly improve overall survival among patients with LCNEC (Figure 2). The median overall survival (mOS) for patients who received adjuvant chemotherapy was 67.0 months, compared to 17.8 months for those who did not receive it (*p* = 0.0011). Analysis of subgroups demonstrated that the survival benefit associated with adjuvant chemotherapy was consistent across categories, including gender, age under 59 years, stage I and stages II–III, smoking status, peripheral tumor location, TFF-1 positivity and negativity, CD56 positivity, and lobectomy. In each subgroup, patients treated with adjuvant chemotherapy exhibited a statistically longer median overall survival than those who did not receive such treatment (Table 6).

Upon the conclusion of this study, 21 individuals with large-cell carcinoma (LCC) (53.8%) and 19 individuals with large-cell neuroendocrine carcinoma (LCNEC) (47.5%) remained alive.

## 4. Discussion

This study was conducted to evaluate the prognostic impact of clinicopathological and immunohistochemical features in patients with resected pulmonary large-cell carcinoma (LCC) and large-cell neuroendocrine carcinoma (LCNEC); it was also intended to clarify the role of adjuvant chemotherapy in these rare tumor types. Given the histopathological and clinical distinctions between LCC and LCNEC, the two groups were analyzed separately to better capture their prognostic heterogeneity.

Our analysis demonstrated that in LCC, age above 61 years, stage II–III disease, and chromogranin A positivity were independent adverse prognostic factors for overall survival, whereas CD56 expression was associated with shorter disease-free survival. These findings are consistent with previous reports suggesting that neuroendocrine differentiation—identified by the presence of markers such as chromogranin A, synaptophysin, and CD56—is associated with unfavorable outcomes in LCC. Harada et al. reported that patients with neuroendocrine-differentiated LCC had poorer survival compared to those with pure LCC [2], and Iyoda et al. also confirmed shorter overall and disease-free survival in LCC patients exhibiting neuroendocrine differentiation [9]. In our cohort, chromogranin A positivity reduced overall survival, while CD56 positivity shortened DFS, supporting the prognostic relevance of these markers.

With respect to treatment, our study found that adjuvant chemotherapy did not significantly affect survival in the overall LCC cohort but conferred a substantial benefit to patients with stage II–III disease. Patients in this subgroup who received adjuvant chemotherapy achieved markedly better survival compared to those treated with surgery alone. This observation is in line with large-scale registry analyses. Hu et al. reported that chemotherapy improved outcomes in LCC patients with stage II or above [10], while another SEER-based study also demonstrated a survival benefit in early-stage but high-risk cases [11]. Taken together, these data reinforce the notion that adjuvant chemotherapy should be considered standard for stage II–III LCC, while its role in stage I remains uncertain.

Meanwhile, LCNEC patients exhibited a distinctly poor prognosis compared to LCC, with a median overall survival of 34 months and a recurrence rate of 60%; most of these recurrences were distant metastases. These findings are consistent with previous reports by Eichhorn et al., who found a median OS of 31.4 months in resected LCNEC [12], and by Kim et al., who documented recurrence in nearly half of their surgically treated LCNEC patients, predominantly at distant sites [13]. The aggressive biology of LCNEC, characterized by rapid progression and early dissemination, underlines its clinical similarity to small-cell lung cancer rather than conventional NSCLC [5].

One of the most striking observations of our study was the consistent survival benefit of adjuvant chemotherapy in LCNEC across all disease stages, including stage I. Stage I patients receiving adjuvant chemotherapy had substantially better survival than those managed with surgery alone. This finding aligns with Veronesi et al., who reported a three-year survival rate of 100% for stage I LCNEC patients treated with adjuvant chemotherapy versus 58% for those without it [14] and Filosso et al., who also confirmed the benefit of adjuvant chemotherapy across stages I–III [15]. While Kim et al. observed limited benefit of adjuvant chemotherapy in stage I, our data add to the growing body of evidence suggesting that systemic therapy should be considered even in early-stage LCNEC [13]. These findings, combined with prior studies, support the notions that LCNEC should be approached more like small-cell lung cancer in terms of systemic therapy and that platinum-based adjuvant regimens may be justified across all stages.

In terms of immunohistochemical markers, synaptophysin positivity was identified as an adverse prognostic factor for overall survival in LCNEC patients. This is partly consistent with prior studies. Eichhorn et al. found high expression rates for CD56, synaptophysin, and chromogranin A in LCNEC, with CD56 and chromogranin A positivity being linked to poorer prognosis [12]. Our findings suggest that synaptophysin may also hold prognostic value, although further validation is warranted.

All adjuvant chemotherapy regimens in our cohort were platinum-based, most commonly cisplatin/etoposide, which reflects current practice patterns. Sarkaria et al. demonstrated that platinum-based regimens resulted in superior survival compared to non-platinum regimens in LCNEC [16]. Therefore, our findings support the continued use of platinum combinations as the backbone of adjuvant therapy in these tumors.

In contrast to large-cell carcinoma (LCC) and large-cell neuroendocrine carcinoma (LCNEC), which currently lack well-established molecularly targeted approaches and are mainly treated with chemotherapy-based strategies, non-small-cell lung cancer (particularly the adenocarcinoma subtype) with oncogenic driver mutations such as EGFR represents a distinct clinical entity with highly effective targeted treatment options. The introduction of EGFR tyrosine kinase inhibitors has markedly improved survival outcomes in this subgroup [17,18]. In rare histologies such as LCC and LCNEC, however, the absence of well-defined actionable targets underscores the urgent need for further research to identify novel therapeutic strategies for large-cell tumors.

This study has several limitations. First, its retrospective design carries the risk of selection bias, particularly with respect to treatment allocation. Second, the sample size, although relatively large for such rare histologies, is limited, thereby reducing statistical power and resulting in wide confidence intervals for some estimates. Post hoc power analysis indicated adequate power for detecting the benefit of adjuvant chemotherapy in LCNEC but limited power in the stage II–III LCC subgroup. Third, immunohistochemical results were extracted from pathology reports without centralized review, which potentially introduced inter-observer variability. Fourth, treatment decisions, including chemotherapy regimen choice and the use of radiotherapy, were determined at the discretion of treating physicians rather than according to standardized protocols, which introduced heterogeneity. Finally, as this study was conducted in a limited number of centers within a single region, the generalizability of the results to other populations may be restricted. These limitations highlight the need for the validation of our results through larger, multicenter prospective trials.

In conclusion, our study underscores the prognostic importance of stage and neuroendocrine differentiation in LCC and LCNEC while providing evidence that adjuvant chemotherapy confers a significant survival benefit in stage II–III LCC and across all stages of LCNEC. The observation of benefit in stage I LCNEC is particularly noteworthy and suggests that systemic therapy should be strongly considered even in early disease. These findings add to the growing body of evidence supporting the integration of adjuvant chemotherapy into the management of LCNEC and may help guide future clinical decision-making and guideline development.

## 5. Conclusions

In this study, disease stage emerged as the principal prognostic determinant in both large-cell carcinoma (LCC) and large-cell neuroendocrine carcinoma (LCNEC). Neuroendocrine markers also demonstrated prognostic relevance, influencing overall and disease-free survival in these groups. Adjuvant chemotherapy provided a survival advantage in stage II–III LCC, whereas in LCNEC, this advantage appeared independent of stage, which underscores adjuvant chemotherapy’s potential role across all disease stages. Furthermore, CD56 expression in LCC was associated with inferior disease-free survival, while synaptophysin positivity in LCNEC correlated with worse overall survival despite adjuvant chemotherapy.

Both LCC and LCNEC exhibit aggressive biological behaviors even when detected at an early stage. In LCC, neuroendocrine marker expression appears to be associated with adverse outcomes, whereas in LCNEC, our findings suggest that systemic therapy could provide a benefit even in stage I disease. These findings highlight the heterogeneity of prognostic factors across these rare NSCLC subtypes and emphasize the need for larger multicenter prospective studies to validate our results and better inform treatment strategies

## Figures and Tables

**Table 1:** Clinicopathological Features of LCC and LCNEC Patients.

Characteristics	Large-Cell Carcinoma n (%)	Large-Cell Neuroendocrine Carcinoma n (%)
Age (years) *	61 (25–76)	58 (43–81)
**Gender**		
Male	33 (84.6)	35 (87.5)
Female	6 (15.4)	5 (12.5)
**Smoking History**		
None	12 (30.8)	4 (10)
Ex-smoker	21 (53.8)	20 (50)
Current smoker	6 (15.4)	16 (40)
Tumor Size (cm) *	3.6 (1.2–6.2)	3.9 (1.2–4.9)
**Pathological lymph node**	23 (58.9)	21 (52.5)
N0	23 (58.9)	21 (52.5)
N1	10 (25.7)	11 (27.5)
N2	6 (15.4)	8 (20)
**Stage**		
I	15 (38.5)	16 (40)
II–III	24 (61.5)	24 (60)
**TNM Stage**		
IAI	1 (2.6)	3 (7.5)
IAII	4 (10.3)	4 (10)
IAIII	3 (7.7)	4 (10)
IB	7 (17.9)	5 (12.5)
IIA	8 (20.5)	5 (12.5)
IIB	10 (25.6)	11 (27.5)
IIIA	4 (10.3)	8 (20)
IIIB	2 (5.1)	0 (0)
**Tumor Localization**		
Central	11 (28.2)	18 (45)
Peripheral	28 (71.8)	22 (55)
**TFF-1**		
Positive	7 (17.9)	13 (32.5)
Negative	32 (82.1)	27 (67.5)
**P40**		
Positive	12 (30.8)	2 (5)
Negative	27 (69.2)	38 (95)
**Synaptophysin**		
Positive	10 (25.6)	26 (65)
Negative	29 (74.4)	15 (35)
**Chromogranin A**		
Positive	4 (10.3)	19 (47.5)
Negative	35 (89.7)	21 (52.5)
**CD56**		
Positive	12 (30.8)	28 (70)
Negative	27 (69.2)	12 (30)

* Age and tumor size were presented as median values.

**Table 2:** Treatment Characteristics of LCC and LCNEC Patients.

	Large-Cell Carcinoma n %	Large-Cell Neuroendocrine Carcinoma n %
**Curative Surgery**	**39 (100)**	**40 (100)**
Lobectomy	28 (71.8)	34 (85)
Pneumonectomy	11 (28.2)	6 (15)
**Adjuvant Chemotherapy**	**22 (56.4)**	**29 (72.5)**
Cisplatin-Vinorelbine	19 (86.4)	1 (3.4)
Carboplatin-Paclitaxel	2 (9.1)	0
Carboplatin-Vinorelbine	1 (4.5)	0
Cisplatin-Etoposide	0	17 (77.2)
Carboplatin-Etoposide	0	10
Carboplatin- Gemcitabine	0	1 (3.4)
**Adjuvant Radiotherapy**	**4 (10.3)**	**9 (22.5)**
**Relapse After Adjuvant Treatments**	**19 (48.7)**	**24 (60)**
Local Recurrence	4	4
Distant Organ Metastasis	15	20
**Post-Relapse Treatment**		
1st Line Chemotherapy	8	19
Radiotherapy	5	2
Surgery	4	2
Supportive Care	2	1
**1st Line Chemotherapy**		
Carboplatin-Pemetrexed	4	0
Carboplatin-Paclitaxel	2	0
Docetaxel	2	0
Platin-Etoposide	0	14
Cisplatin-Gemcitabine	0	1
Irinotecan	0	1
Topotecan	0	2
CAVI	0	1

n: number, %: percentage, CAVI: Doxorubicin-Cyclophosphamide-Vincristine.

**Table 3:** Univariate and multivariate analysis of factors affecting overall survival in LCC Patients.

Large-Cell Carcinoma							
		**mOS** **(Months)**	**Univariate** **(*p* Value)**	**Multivariate** ***p* Value** **(HR)**	mDFS (Months)	Univariate (*p* Value)	Multivariate *p* Value (HR)
Gender	Male	80.1	0.4		64.1	0.32	
	Female	NR			NR		
Age	<61	NR	**0.020**	**0.047 (HR: 0.279)**	NR	0.065	
	≥61	62.029			53.06		
Smoking	Yes	80.1	0.56		77.1	0.53	
	No	NR			NR		
Stage	I	NR	**0.028**	**0.016 (HR: 0.198)**	NR	**0.02**	**0.022 (HR: 0.26)**
	II–III	62.02			47.2		
Tumor Localization	Central	65.8	0.49		66.1	0.74	
	Peripheral	80			77.14		
TTF-1	Positive	NR	0.3		NR	0.26	
	Negative	65.873			64.1		
P40	Positive	62.0	0.73		50.1	0.41	
	Negative	80.1			77.1		
Synaptophysin	Positive	NR	0.81		45.8	0.42	
	Negative	79.6			91.1		
Chromogranin A	Positive	52.3	**0.017**	**0.011** **(HR: 0.088)**	**43.1**	0.056	
	Negative	94.9			122.3		
CD56	Positive	65.3	0.15		32.7	**0.043**	**0.034 (HR: 0.35)**
	Negative	83.8			91.1		
Surgical Procedure	Lobectomy	92.8	0.35		91.1	0.64	
	Pneumonectomy	62.0			53.0		
Adjuvant Chemotherapy	Yes	94.9	0.70		77.1	0.73	
	No	65.8			64.1		
Adjuvant Radiotherapy	Yes	36.5	0.96		34.3	0.86	
	No	80.1			77.1		

NR: Not Reached; HR: Hazard Ratio.

**Table 4:** The Association of Adjuvant Chemotherapy with Survival According to Subgroups of LCC Patients.

		Adjuvant Chemotherapy Yes		Adjuvant Chemotherapy No		
		n	mOS	n	mOS	*p*
Gender	Female	2	NR	4	NR	0.85
	Male	20	94.9	13	62.0	0.46
Age	<61	8	NR	6	NR	0.39
	≥61	14	66.8	11	70.2	0.97
Stage	I	3	NR	12	NR	0.37
	II–III	19	80.1	5	32.7	**0.009**
Smoking	Yes	18	80.1	9	NR	0.669
	No	4	NR	8	NR	0.107
Tumor Localization	Central	7	94.7	4	62.0	0.206
	Peripheral	15	80.1	13	NR	0.87
TTF-1	Positive	3	NR	4	NR	0.18
	Negative	19	80.1	13	65.8	0.95
P40	Positive	9	94.9	3	62.0	0.88
	Negative	13	80.1	14	65.8	0.66
Synaptophysin	Positive	8	NR	2	52.3	0.810
	Negative	14	94.9	15	65.8	0.609
Chromogranin A	Positive	3	52.3	1	41.6	0.56
	Negative	19	65.8	16	94.9	0.67
CD56	Positive	7	41.0	5	53.8	0.38
	Negative	15	94.9	12	NR	0.926
Surgical Procedure	Lobectomy	14	62.0	14	52.3	0.86
	Pneumonectomy	8	94.9	3	65.8	0.181

n: number; mOS: median overall survival; NR: Not Reached.

**Table 5:** Univariate and Multivariate Analysis of Factors Affecting Overall Survival in LCNEC Patients.

Large-Cell Neuroendocrine Carcinoma							
		mOS (Months)	Univariate *p*	Multivariate *p* (HR)	mDFS (Months)	Univariate *p*	Multivariate *p* (HR)
Gender	Male	28.02	0.27		24.1	0.49	
	Female	NR			61.9		
Age	<58	28.3	0.78		26.7	0.93	
	≥58	41.2			31.3		
Smoking	Yes	28.3	0.48		26.7	0.37	
	No	41.5			36.0		
Stage	I	NR	**0.001**	**0.017 (HR: 0.20)**	NR	**0.001**	**0.005 (HR: 0.20)**
	II–III	24.0			15.0		
Tumor Localization	Central	28.0	0.32		15.4	0.064	
	Peripheral	41.5			35.8		
TTF-1	Positive	41.5	0.55		29.8	0.46	
	Negative	24.8			17.6		
Synaptophysin	Positive	24.8	**0.014**	**0.037 (HR: 0.30)**	17.6	0.057	
	Negative	39.4			61.9		
Chromogranin A	Positive	21.7	**0.028**	0.14	14.8	0.077	
	Negative	66.9			40.0		
CD56	Positive	23.0	0.056		35.2	0.087	
	Negative	NR			14.4		
Surgical Procedure	Lobectomy	66.9	**0.001**	**0.048 (HR: 0.31)**	36.0	**0.002**	0.57
	Pneumonectomy	17.8			13.5		
Adjuvant Chemotherapy	Yes	67.0	**0.0011**	**0.030 (HR: 0.264)**	61.9	**0.001**	**0.02 (HR: 0.211)**
	No	17.8			12.9		
Adjuvant Radiotherapy	Yes	36.8	0.95		26.7	0.47	
	No	28.3			31.3		

NR: Not Reached, HR: Hazard Ratio.

**Table 6:** The Association of Adjuvant Chemotherapy with Survival According to Subgroups of LCNEC Patients.

		Adjuvant Chemotherapy Yes		Adjuvant Chemotherapy No		
		n	mOS	n	mOS	*p*
Gender	Female	4	64.6	1	21.7	**0.046**
	Male	25	34.2	10	15.9	**0.007**
Age	<58	15	NR	6	15.2	**0.006**
	≥58	14	41.5	5	21.7	0.055
Stage	I	12	NR	4	21.7	**0.038**
	II–III	17	28.0	7	15.2	**0.004**
Smoking	Yes	25	66.9	11	17.8	**0.002**
	No	4	41.5	0	-	-
Tumor Localization	Central	12	28.3	6	14.7	0.33
	Peripheral	17	NR	5	17.08	**0.001**
TTF-1	Positive	11	41.5	2	17.8	**0.016**
	Negative	21	66.9	9	15.9	**0.008**
Synaptophysin	Positive	17	28.3	9	17.8	0.051
	Negative	12	NR	2	12.9	0.33
Chromogranin A	Positive	10	21.7	9	15.9	**0.05**
	Negative	19	28.0	2	16.8	0.42
CD56	Positive	18	41.5	10	15.9	**0.01**
	Negative	10	23.5	1	-	0.46
Surgical Procedure	Lobectomy	26	66.9	8	18.0	**0.018**
	Pneumonectomy	3	22.3	3	15.2	0.55

n: number; mOS: median overall survival; NR: Not Reached.

## Data Availability

For data request or enquiries regarding this study, Doğan Bayram (drdoganb@gmail.com) can be contacted.
